# Vulnerable Myocardial Interstitium in Patients With Isolated Left Ventricular Hypertrophy and Sudden Cardiac Death: A Postmortem Histological Evaluation

**DOI:** 10.1161/JAHA.112.001511

**Published:** 2012-06-22

**Authors:** Balaji K. Tamarappoo, Benjamin T. John, Kyndaron Reinier, Carmen Teodorescu, Audrey Uy-Evanado, Karen Gunson, Jonathan Jui, Sumeet S. Chugh

**Affiliations:** Heart Institute, Cedars-Sinai Medical Center, Los Angeles, CA (B.K.T., B.T.J., K.R., C.T., A.U.-E., S.S.C.); Department of Pathology, Oregon Health and Science University, Portland, OR (K.G.); Department of Emergency Medicine, Oregon Health and Science University, Portland, OR (J.J.); Cleveland Clinic Foundation, Cleveland OH (B.K.T.); Vancouver Clinic, Vancouver, WA (B.T.J)

**Keywords:** death, sudden, collagen, hypertrophy, myocardium, remodeling

## Abstract

**Background:**

Concentric left ventricular hypertrophy (LVH) is independently associated with increased risk of sudden cardiac death (SCD). Some animal models of LVH display specific alterations of the myocardial interstitium that could increase myocardial vulnerability to ventricular arrhythmias, but these merit evaluation in humans with LVH and SCD.

**Methods and Results:**

Twelve consecutive patients with isolated LVH and SCD (LVH+SCD) in the absence of hypertrophic cardiomyopathy, coronary disease, or other cardiac structural abnormality were ascertained in the Oregon Sudden Unexpected Death Study. Detailed postmortem comparisons were conducted with 18 controls who had isolated LVH and unnatural deaths (Control Group A) and 6 controls who had structurally normal hearts and unnatural deaths (Control Group B). Postmortem left ventricular myocardial sections were obtained for measurement of collagen volume fraction, characterization of gap junctions, and quantification of collagen subtypes. Heart weight normalized to body weight was higher in LVH+SCD cases (6.9±1.2 g/kg) than in Control Group A (5.3±1.4 g/kg) and Control Group B (4.2±0.3 g/kg); *P*=0.001. Collagen volume fraction was also higher in LVH+SCD cases (3.1±0.4) than in Control Group A (2.3±0.4) and Control Group B (1.6±0.3); *P*=0.0002. The relative amount of collagen III was significantly higher in LVH+SCD cases (33.0±4.4%) than in Control Group A (20.9±4.3%) and Control Group B (13.4±3.5%); *P*=0.0001. There was an overall increase in the number of connexin 43–labeled gap junctions with increasing myocyte size. No subject was found to have high-risk hypertrophic cardiomyopathy mutations.

**Conclusions:**

In addition to the expected increase in myocardial mass and overall collagen content, SCD with isolated LVH was associated with relative abundance of type III collagen, a novel finding that warrants further mechanistic evaluation. **(*J Am Heart Assoc*. 2012;1:e001511 doi: 10.1161/JAHA.111.001511.)**

## Introduction

Left ventricular hypertrophy (LVH) is an independent risk factor for sudden cardiac death (SCD),^1–3^ a major cause of death in the United States and around the globe.^4,5^ The increased risk of SCD in LVH is due to increased propensity for ventricular arrhythmias, and we have recently reported the likely existence of distinct pathways that increase risk of arrhythmogenesis with LVH.^2^ The elucidation of these pathways is likely to be an important step for developing enhanced methods of prediction, prevention, and therapeutics in patients with LVH. Because the prevalence estimates of LVH in the general population are as high as 16%,^6,7^ the public health implications are significant.^4,5,8^

Although LVH is associated with several pathological changes in the left ventricular (LV) myocardium that may predispose to ventricular arrhythmogenesis,^9–14^ an important aspect is myocardial fibrosis.^12,15–17^ Myocardial fibrosis can impair the electrical coupling of cardiomyocytes by separating myocytes with extracellular matrix (ECM) proteins, and its presence in LVH may create a substrate of tissue heterogeneity from which reentrant tachyarrhythmias could arise.^18,19^ We have previously reported global myocardial interstitial remodeling in subjects with idiopathic myocardial fibrosis and SCD.^20^ Although experimental animal models of hypertrophy have demonstrated both an accumulation of collagen in the myocardium and an alteration in the relative abundance of collagen subtypes type I and III,^21–23^ these phenomena need to be evaluated in humans with LVH and SCD, especially with comparison to patients who have LVH and no relationship to SCD (ie, unnatural death). In addition, gap junctions play an integral role in the conductance properties of the myocardium, and alterations in the number and density of connexin 43–labeled gap junctions at the intercalated disc have been reported in human LVH.^24^ We characterized the nature and extent of interstitial remodeling in a postmortem evaluation of patients with isolated LVH and SCD, including alterations in the expression of collagen types I and III, as well as distribution of connexin 43–labeled gap junctions. We also screened all cases and controls with LVH for high-risk hypertrophic cardiomyopathy mutations.

## Methods

### Ascertainment of Subjects

The Institutional Review Board at the Oregon Health and Science University approved all aspects of this investigation. Residents of Multnomah, Clackamas, and Washington counties, Oregon, who had SCD between February 1, 2002, and June 30, 2005, were identified through multiple sources (the emergency medical response system, the Medical Examiner's office, and 16 area hospitals) as part of an ongoing population-based study of sudden unexpected death (the Oregon Sudden Unexpected Death Study). All cases of SCD from the Portland, Oregon, metropolitan area included in the analysis, as well as the 2 control groups, were required to have a detailed postmortem exam and evaluation by the Medical Examiner's office. At the time of autopsy, full-thickness tissue blocks were obtained from the LV free wall and stored separately in 10% neutral buffered formalin at room temperature and unfixed at −80°C.

### Definition of SCD

SCD was defined as an unexpected death within 1 hour of symptom onset (witnessed) or an unexpected death in which the individual had been observed alive and symptom free 24 hours previously (unwitnessed). If any nonarrhythmic cause of sudden death was discovered at autopsy, the case was excluded.

### Definition of Isolated LVH

Isolated LVH was defined as a heart weight greater than the 95% upper limit of normal when normalized by body weight and sex.^25,26^ There had to be an absence of significant coronary artery disease (vessel stenosis >50% or evidence of acute or chronic myocardial infarction), valvular disease, or congenital heart disease, and the hypertrophy had to be without evidence of myocardial disarray on histological exam.

### Identification of Cases and Controls

**Figure 1.**
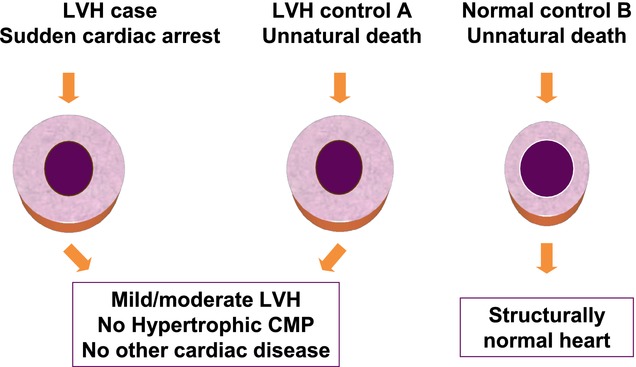
Illustration of the postmortem human model with myocardial sections compared among cases (LVH and sudden cardiac arrest), Control Group A (LVH and unnatural death), and Control Group B (normal controls with unnatural death). CMP indicates cardiomyopathy.

Cases (LVH+SCD, n=12) were defined as individuals who sustained SCD with evidence of isolated LVH on postmortem exam and without evidence of extracardiac findings that could have contributed to their demise. During the same time period and from the same geographical area, a control group was assembled of individuals who were found to have isolated LVH but who sustained a noncardiac, unnatural death due to conditions such as suicide or trauma (Control Group A, n=18). A second non-LVH control group consisted of individuals who were found to have a normal cardiac exam and sustained a noncardiac, unnatural death (Control Group B, n=6) (Figure 1).

### Screening of Cases and Controls for Mutations in Exons 8, 9, and 11 of *Troponin T* and Exons 13, 14, and 19 of *β-Myosin Heavy Chain*

To ensure that individuals with LVH had isolated myocardial hypertrophy in the absence of sarcomeric gene mutations that are known to be associated with SCD in hypertrophic cardiomyopathy, we screened all patients in LVH+SCD and Control Group A for high-risk hypertrophic cardiomyopathy mutations.^27–29^ Accordingly, we sequenced exons 13, 14, and 19 of the *β-myosin heavy chain (βMHC)* gene and exons 8, 9, and 10 of the *troponin T (TnT)* gene.^27–29^ Amplification of the exon sequences of interest for *βMHC* and *TnT* was performed with allele-specific oligonucleotide probes. In brief, DNA for amplification by polymerase chain reaction was extracted from blood leukocytes. Exons 13, 14, and 19 of the *βMHC* gene^27^ and exons 8, 9, and 10 of the *TnT* gene were amplified by polymerase chain reaction as described previously.^29^ Automated sequencing was performed, and DNA sequences were analyzed.

### Evaluation of ECM Collagen Composition

#### Collagen Volume Fraction

Formalin-fixed tissue was processed and embedded in paraffin with the use of standard histological techniques. Myocardial sections, 5 μm thick, were stained with Picrosirius Red, and birefringent collagen fibrils were viewed under polarized light with a 40× objective.^20^ Each section was divided into 4 quadrants, and 4 fields were selected randomly within each quadrant. A digitized gray-scale image of the collagen fibrils was obtained, and the area encompassed by the collagen fibrils was determined (ImageTool Version 2.0, Houston, TX). This step was repeated to determine the area of the background myocardium. Percent collagen volume fraction (CVF) was calculated by dividing the area of collagen fibrils by the sum total area of collagen fibrils and myocardium. CVF for each myocardial section was expressed as the average of 16 fields. All sections were stained in batches of case and respective controls. CVF analysis was performed in a randomized, blinded fashion. The intraobserver and interobserver correlations for this method are 0.91 and 0.89, respectively.

#### Type I and Type III Collagen Distribution

Immunohistochemical staining for collagen type I and type III was performed in a subset of tissue samples from LVH+SCD cases, Control Group A, and Control Group B, and semiquantitative measurement of the relative amounts of collagen type I and type III was performed. Fresh, frozen tissue blocks were embedded in Optimal Cutting Temperature medium, and 10-μm sections were obtained. A mouse anti-human type I collagen antibody (1:500 dilution, clone I-8H5; ICN Biomedicals, Inc, Aurora, OH) and a rabbit anti-human type III collagen antibody (1:500 dilution; Biodesign International, Saco, ME) were applied to each section separately for 1 hour at room temperature. A mixture of anti-mouse fluorescein isothiocyanate conjugate (1:200, Sigma-Aldrich, Inc, Saint Louis, MO) and anti-rabbit Alexa 594 conjugate (1:2000, Molecular Probes, Inc, Eugene, OR) was then applied to each section for 1 hour. All sections were stained with 0.3% (w/v) Sudan Black in 70% ethanol to quench autofluorescence and were mounted in Gel Mount (Sigma-Aldrich, Inc, Saint Louis, MO). Immunostaining was performed in batches to include cases and respective controls and was blinded and randomized before evaluation.

All sections were analyzed with the use of an API DeltaVision wide-field, optical sectioning microscope and image analysis system (Applied Precision, LLC, Issaquah, WA). With a 60× objective, 5 random fields per section were imaged. Ten optical sections (0.5 μm thick) were taken in each filter for the fluorescein and Alexa 594 labels. Each optical section underwent deconvolution processing according to the iterative constrained algorithm of Sedat and Agard to reduce out-of-focus blur. Gray-scale images of each label were then obtained by compressing the 10 deconvoluted images. Areas of fluorescent-labeled type I and type III collagen were obtained from each of their respective gray compressed images. Areas of background myocardium were obtained, and ratios of type I and type III collagen to background were calculated. The type I : type III collagen ratio was then calculated from these values and presented as an average of 5 fields per subject.

#### Collagen Extraction and Quantification

The purification, digestion of collagen with cyanogen bromide (CNBr), separation of type I and type III collagen by sodium dodecyl sulfate polyacrylamide gel electrophoresis (SDS-PAGE), and quantification by gel scanning was performed as described by Mukherjee and Sen.^30^ Type I collagen and type III collagen were separated by SDS-PAGE on a 10% to 20% polyacrylamide gradient gel (Biorad, Hercules, CA) stained with 5% Coomassie Blue-250. Intensities of the Coomassie-stained protein bands were measured by densitometry. The type I : type III collagen ratio was given by the ratio of the intensity of collagen I–band H (CNBr digest fragment 8) and collagen III–band M (CNBr fragments 5 and 9).^30^

### Evaluation of Connexin 43–Labeled Gap Junction Plaques

#### Gap Junction Plaque Morphometry

Frozen tissue blocks of LV myocardium were embedded in Optimal Cutting Temperature medium, and 10-μm sections were obtained. A mouse anti-human connexin 43 antibody (1:500, clone P4G9, Fred Hutchinson Cancer Research Center, Seattle, WA) was applied to each section for 1 hour at room temperature. Anti-mouse fluorescein conjugate (1:200, Sigma-Aldrich, Inc, Saint Louis, MO) was then applied for 1 hour at room temperature. All sections were counterstained with 0.3% Sudan Black and mounted as described previously. Immunostaining was performed in batches to include cases and respective controls and was blinded and randomized before evaluation.

With the use of the API DeltaVision wide-field, optical sectioning microscope, 5 to 7 intercalated discs between myocytes were identified *en face*. Optical sections were imaged at 0.2-μm thickness through the intercalated disc to capture all labeled gap junction plaques within this disc. All optical sections underwent deconvolution processing as described previously, and a compressed image was obtained. The length (μm) and area (μm^2^) of each gap junction plaque and the cross-sectional area (CSA; μm^2^) of each intercalated disc were measured from the compressed image and presented as an average of 5 to 7 discs. Total surface area (TSA) of gap junction plaques (μm^2^) in each intercalated disc was obtained by adding the individual gap junction plaque areas for each intercalated disc. Percent of gap junction plaque area per intercalated disc was calculated by dividing the TSA of gap junction plaques by the CSA of the respective intercalated disc. The gap junction plaque density (μm^2^/μm^3^) was calculated by dividing the TSA of gap junction plaques by the total volume of intercalated disc imaged (measured CSA of intercalated disc multiplied by thickness of intercalated disc).

### Statistical Analysis

All continuous data are reported as mean ± standard deviation. Comparisons between the 3 independent patient groups (case group [LVH+SCD] and Control Groups A and B) were performed with the Kruskal-Wallis test to test for the significance of the difference among the distributions (level of significance *P*<0.05). Age and sex adjustment was performed by nonparametric regression analysis. The Wilcoxon rank-sum test was used to compare the distribution between any 2 independent patient groups. Spearman's method was used to assess correlations between measured values.

## Results

### Patient Characteristics

Characteristics of all subjects are shown in Table 1. The case group (LVH+SCD) consisted of 12 individuals, 2 female and 10 male, with a median age of 41 years. Control Group A (LVH and unnatural death) consisted of 18 individuals, 5 female and 13 male, with a median age of 46 years. Control group B (normal controls) consisted of 6 individuals, 3 female and 3 male, with a median age of 39 years. There was no significant difference in age between the 3 groups (*P***=**0.21).

**Table 1.** Characteristics of Study Subjects

Age/Sex	Heart Weight, g	Body Weight, kg	Heart Weight : Body Weight
Control Group A (LVH + non-SCD)			

44/Male	600	113	5.31

33/Male	460	136	3.38

51/Female	440	127	3.46

54/Female	400	63	6.35

48/Male	610	90	6.78

50/Male	560	113	4.96

41/Male	550	127	4.33

37/Female	470	181	2.59

41/Male	600	81	6.33

39/Male	490	81	6.05

24/Male	440	64	6.88

41/Female	380	52	7.31

47/Female	500	106	4.72

56/Male	500	93	5.38

49/Male	650	98	6.63

49/Male	510	90	5.67

35/Male	590	165	3.58

45/Male	510	81	6.29

Control group B (normal controls)			

34/Male	440	104	4.23

50/Female	300	77	3.89

35/Female	300	72	4.17

29/Female	180	45	4

43/Male	320	79	4.05

20/Male	375	77	4.87

Cases (LVH+SCD)			

51/Male	710	125	5.68

41/Male	520	79	6.58

50/Male	830	129	6.43

47/Male	800	91	8.79

18/Male	440	66	6.67

41/Male	370	46	8.04

63/Male	880	138	6.38

40/Female	430	56	7.68

30/Male	665	103	6.46

23/Female	750	148	5.07

49/Male	510	83	6.14

48/Male	870	95	9.16

### Heart Weight : Body Weight Ratios

There was a significant difference in the heart weight : body weight ratios between the individual groups of patients analyzed in the study (*P***=**0.001; age- and sex-adjusted *P***=**0.002). There was a significant difference in the heart weight : body weight ratios between cases and Control Group A (6.9±1.2 versus 5.3±1.4 g/kg; *P*=0.01) and between cases and Control Group B (6.9±1.2 versus 4.2±0.3 g/kg; *P*=0.004). Although the heart weight : body weight ratio of individuals with LVH and death from noncardiac causes (Control Group A) was higher than that of non-LVH controls (Control Group B) (5.3±1.4 versus 4.2±0.3 g/kg), the difference was not statistically significant (*P***=**0.10).

### Sequence Analysis

There were no mutations in the coding sequence in exons 8, 9, and 11 of the *TnT* gene or in exons 13, 14, and 19 of the *βMHC* gene in cases with LVH+SCD or in Control Group A when compared to the wild-type coding sequence.

### ECM Collagen Composition

#### Collagen Volume Fraction

**Figure 2.**
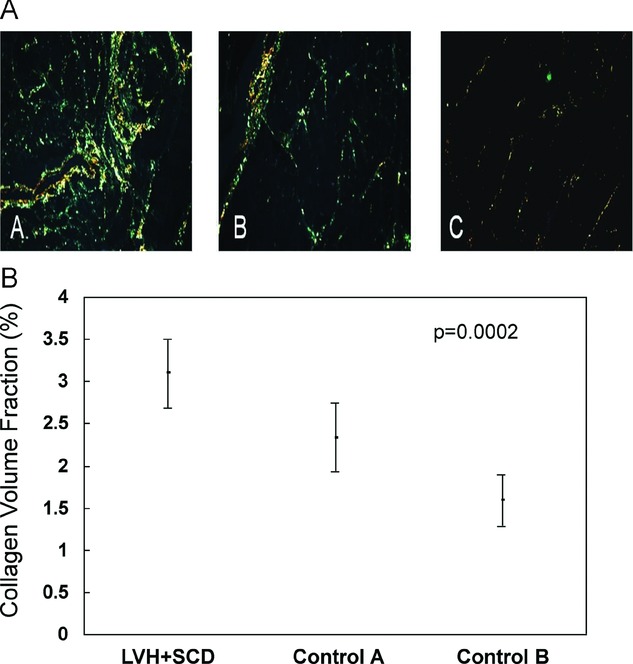
**A,** Myocardial sections showing collagen fibrils stained with Picrosirius Red and viewed under polarized light (40×). Stain A shows a representative section from an individual with LVH+SCD. Stains B and C show representative sections from Control Groups A (LVH + non-SCD) and B (normal control), respectively, with significantly fewer collagen fibrils detectable in the interstitium. **B,** CVF was quantified as percentage of myocardium comprised of collagen fibrils per high-power field. CVF was expressed as mean±SD for individuals with LVH+SCD and controls. Subjects with LVH+SCD had significantly greater CVF (3.1±0.4%) than Control Group A (LVH + non-SCD) and Control Group B (normal control) (*P*=0.006 and *P*=0.007, respectively). There was also a significant difference in mean CVF between the 2 control groups (Control Group A 2.3±0.4% vs Control Group B 1.6±0.3%; *P*=0.02).

CVF was significantly different among the 3 different patient groups: cases 3.1±0.4, Control Group A 2.3±0.4, and Control Group B 1.6±0.3 (*P***=**0.0002; age- and sex-adjusted *P***=**0.0001; Figure 2A and Figure 2B). CVF for cases was different compared to Control Group A (*P***=**0.006) and Control Group B (*P***=**0.007). There was also a significant difference in CVF between the 2 control groups (*P***=**0.02). There was a moderate correlation between CVF and heart weight : body weight ratio (*r*=0.66, *P***=**0.0005).

#### Collagen Subtype Distribution

**Figure 3.**
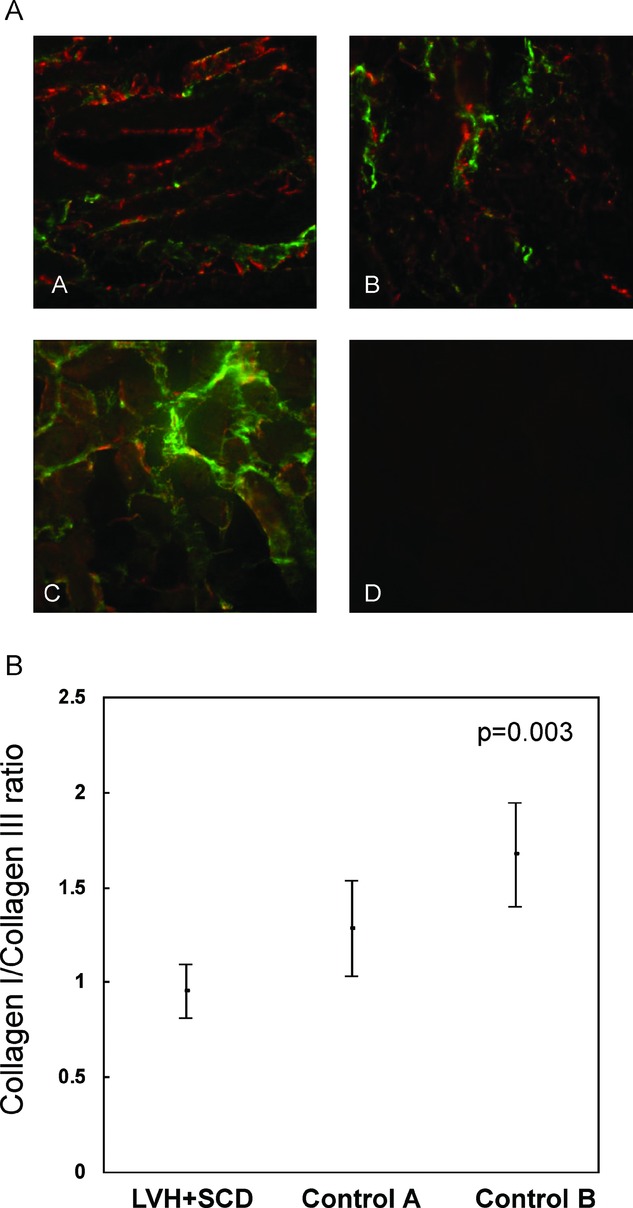
**A,** Comparisons of immunohistochemical staining for collagen type I (Anti-Type I, fluorescein-green) and type III (anti-Type III, Alexa 594-red) in representative cases (LVH+SCD), Control Group A (LVH + non-SCD), and Control Group B (non-LVH controls). Greatest intensity of staining for type III collagen (green) is seen for a case subject in stain A; significantly less intensity of staining is seen for Control Group A subject in stain B, and the least for Control Group B subject in stain C (predominant staining for collagen type I). Stain D represents a negative control with absence of autofluorescence. **B,** Semiquantitative measurement of the relative amounts of collagen type III and type I revealed a significant difference in the relative expression of collagen III and collagen I between cases and controls. The type I : type III collagen ratio was expressed as mean±SD for individuals with LVH+SCD and controls. Cases had a lower mean type I : type III collagen ratio (0.95±0.14) than those of Control Group A (1.28±0.25) and Control Group B (1.67±0.27); *P***=**0.003.

Among the subset of individuals with LVH+SCD whose LV tissue was analyzed by immunohistochemical staining for collagen I and collagen III, there was higher-intensity staining for type III collagen than that observed in Control Group A or Control Group B (Figure 3A). A semiquantitative estimation of the relative amounts of type III and type I collagen in these tissue sections revealed a difference in the relative expression of collagen III and collagen I among cases, Control Group A, and Control Group B (*P***=**0.003; age- and sex-adjusted *P*<0.0001). Cases had a lower mean type I : type III collagen ratio (0.95±0.14) than that of Control Group A (1.28±0.25) and Control Group B (1.67±0.27) (Figure 3B). No significant correlation between the type I : type III ratio and heart weight : body weight ratio was observed (*r*=–0.355, *P*=0.12).

#### Extraction and Quantification of Collagen I and III From LV Myocardium

**Figure 4.**
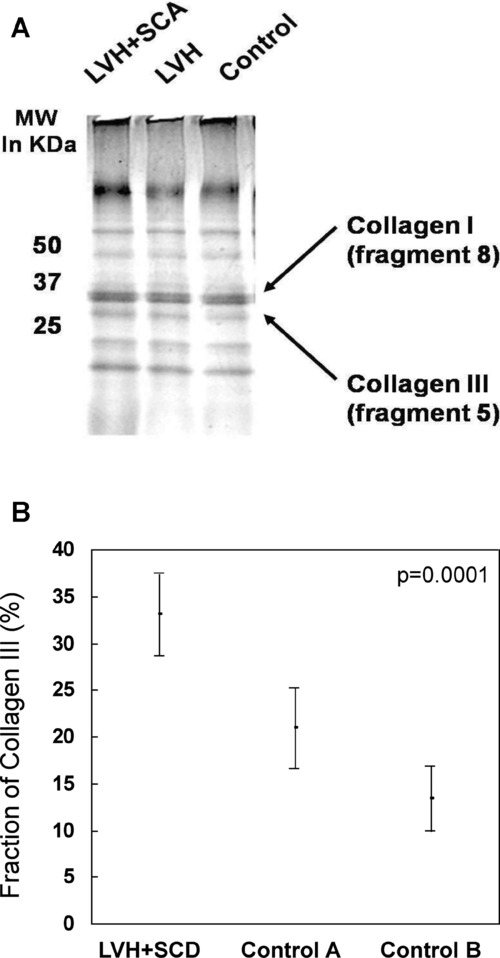
**A,** Collagen III and collagen I were extracted by digestion of 1 g myocardium, separated by SDS-PAGE, and stained with Coomassie Blue-250. Representative gel with collagen I–band H (CNBr digest fragment 8) and collagen III–band M (CNBr fragments 5 and 9) is shown. SCA indicates sudden cardiac arrest; MW, molecular weight. **B,** Intensities of the Coomassie-stained protein bands were measured by densitometry. Relative amounts of type III : type I collagen in the extract were given by the ratio of the intensity of collagen III–band M (CNBr fragments 5 and 9) and collagen I–band H (CNBr digest fragment 8). Percent of collagen III compared to the sum of collagen I and collagen III extracted was expressed as mean±SD for individuals with LVH+SCD and controls. Percent myocardial collagen type III was significantly higher in cases (LVH+SCD; 33±4%) than in Control Group A (LVH + non-SCD; 21±4%; *P***=**0.0001) and Control Group B (non-LVH, non-SCD; 13±3%; *P***=**0.004).

Collagen was extracted from 1 g of tissue per subject, which was removed from the LV encompassing the myocardium of 2 representative regions corresponding to the LV lateral wall and the septum, and densitometry is shown in Figure 4A. Densitometric analysis of the collagen subtypes revealed a significant difference in the abundance of collagen III in the LV myocardium of patients with LVH+SCD (cases), Control Group A, and Control Group B (*P*<0.0001). When expressed as a percentage of the total collagen in the LV myocardium, there was a significant difference in the fraction of collagen type III among the 3 groups of patients (*P***=**0.0001). There was an increase in the fraction of collagen type III in the ECM in individuals with LVH+SCD (33.0±4.4%) when compared to Control Group A (20.9±4.3%; *P***=**0.0001) or Control Group B (13.4±3.5%; *P***=**0.004) (Figure 4B). These results are similar to observations from semiquantitative measurements of immunohistochemical analysis of tissue sections of LV myocardium from these patients, shown in Figure 3.

### Connexin 43–Labeled Gap Junction Plaque Distribution

**Figure 5.**
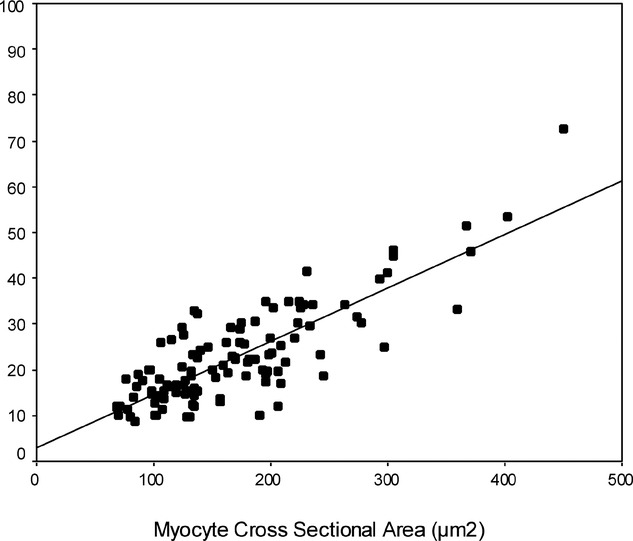
Intercalated discs were identified by microscopy, and gap junctions were identified within intercalated discs by immunostaining of connexin 43. CSAs of intercalated discs and gap junctions were quantified from digitized microscopic images. Gap junction plaque area was compared to myocyte CSA. Increasing myocyte size as measured by CSA is associated with increase in gap junction plaque surface area (*r*=0.821, *P*<0.001).

No significant differences were observed in the individual gap junction plaque length or area between cases and controls (Table 2). Although there was a trend toward greater gap junction plaque TSA in cases versus controls, it was not statistically significant (cases 25.93±2.79 μm^2^ versus Control Group B 21.09±1.39 μm^2^; *P*=0.08). The percentage of the CSA of the intercalated disc occupied by the gap junction plaques remained preserved in cases versus controls, and the density of gap junction plaques in the intercalated disc at the end of each myocyte did not significantly differ between cases and controls. However, when the gap junction plaque surface area in LVH hearts was compared to the myocyte CSA, there was a strong and statistically significant correlation. Increases in myocyte CSA were associated with an increase in connexin 43 gap junction plaque area (*r*=0.821, *P*<0.001; Figure 5).

**Table 2.** Connexin 43–Labeled Gap Junction Distribution

	Gap Junction Disc	Gap Junction Disc	Gap Junction	Gap Junction	Gap Junction Plaque
	Length, μm*	Area, μm^2^†	TSA, μm^2^‡	%Plaque Area¶	Density, μm^2^/μm^3^**
Cases (LVH+SCD) (n=5)	0.517 ± 0.017	0.266 ± 0.026	25.93 ± 2.79	13.4 ± 0.6%	0.0408 ± 0.0034

Control Group A (n=7)	0.520 ± 0.015	0.234 ± 0.012	23.02 ± 1.40	14.7 ± 0.7%	0.0463 ± 0.0043

Control Group B (n=6)	0.519 ± 0.012	0.236 ± 0.010	21.09 ± 1.39	13.5 ± 0.6%	0.0400 ± 0.0024

* † ‡ ¶ ** No significant difference (*P*≥0.08) on comparison of means between Cases vs Control Group A, Cases vs Control Group B, Control Group A vs Control Group B.

## Discussion

We observed significant and specific alterations in the nature of myocardial interstitial remodeling in LVH+SCD that were not identified in LVH controls or normal controls. In addition to greater normalized heart weight adjusted to body weight, there was an increase in the amount of myocardial fibrosis, quantified as a 1.3-fold increase CVF in individuals with LVH+SCD compared to individuals with isolated LVH and noncardiac death. Specifically, this increase in the amount of collagen was associated with an altered distribution in collagen type I and type III, with a significant increase in the relative abundance of collagen type III. There was an overall increase in the number of connexin 43–labeled gap junctions with increasing myocyte size; however, the density of gap junction plaques per intercalated disc did not change with this increase.

A likely strength of this study relates to the design: postmortem access to hearts of subjects who had isolated LVH and SCD and the ability to compare these with hearts from subjects with isolated LVH or normal hearts who had unnatural deaths. Prior autopsy series have demonstrated a prevalence of isolated LVH of ≈18% in subjects who sustain SCD.^25^ An increase in the incidence of ventricular arrhythmias with LVH has long been documented,^31,32^ and LVH is recognized as an independent risk factor for SCD.^1,2^ There also appears to be a continuous and graded increase in the occurrence of ventricular arrhythmias with increasing LV mass,^11,33^ suggesting an association between the extent of hypertrophy and the risk of arrhythmia. Consistent with these earlier studies, we found that individuals with LVH who sustained SCD manifested a greater degree of hypertrophy, defined by heart weight : body weight ratios, compared to a control group of individuals with LVH and non-SCD.

From the mechanistic standpoint, it has been hypothesized that the increase in the ECM creates an anatomic uncoupling of adjacent myocytes such that zigzag and slow electrical propagation may occur, thereby creating potential sites for reentry.^18^ Although increased collagen is expected to be observed in LVH,^34,35^ we detected a significant increase in the amount of collagen in individuals with LVH+SCD compared to the LVH controls and normal control groups, which supports the concept that LVH+SCD subjects were more vulnerable to ventricular arrhythmogenesis due to fibrosis-mediated reentry. More importantly, earlier studies were not designed to make these specific comparisons, which have yielded what could be a novel form of remodeling unique to patients with SCD and isolated LVH. Our observation that there is a preferential increase in type III collagen in the myocardium of individuals with LVH who experience SCD suggests that changes in the composition of the ECM may affect the conduction properties of the myocardium and may create conditions conducive for ventricular arrhythmias. Myocardial collagen consists primarily of type I and type III collagen, and in animal models LVH has been shown to be associated with an early increase in the amount of type I collagen with a resultant alteration in the type I : type III collagen ratio.^23,36^ Previous studies have demonstrated elevated serum markers of both type I and type III collagen in human hypertensive heart disease,^34,37–39^ but there had been a lack of studies identifying the relative tissue distribution of these collagen subtypes in ventricular hypertrophy in humans, especially in the context of SCD. Type III collagen is thought to be the primary isoform that increases within the endomysial weave that surrounds individual myocytes during the development of ventricular hypertrophy,^22^ and an increase in the density of this endomysial weave has been documented by scanning electron microscopy.^34^ Increasing the lateral cell-to-cell contact may result in alterations in the anisotropic properties of the myocardium and may create the conditions favorable for reentry.^40^

Resident fibroblasts normally found in the myocardium are relatively quiescent, but under conditions of myocardial injury or stress they transdifferentiate into myofibroblast cells that are responsible for the enhanced synthesis of ECM proteins, including collagen I, collagen III, and fibronectin.^41–43^ In pathological states such as LVH, there is a dysregulation of myofibroblast removal, and the continued presence of myofibroblasts is thought to result in the sustained synthesis of the ECM proteins and myocardial fibrosis.^44^ It is possible that the abnormal collagen remodeling occurs because of proliferation or abnormal function of myofibroblasts in the LVH interstitium. The high secretory capacity of myofibroblasts may disrupt the normal balance between ECM synthesis and degradation, resulting in a rate of collagen deposition that far outweighs the rate of collagen degradation.^45^

Gap junction organization is an important determinant of normal myocardial conduction, in that it allows low-resistance conduction in a longitudinal orientation. It has been shown that alterations in gap junction distribution can affect the anisotropic properties of the myocardium. Previous studies in humans with decompensated LVH have shown a decrease in the relative density of connexin 43 gap junctions at the intercalated disc.^39^ Our studies demonstrate that the density of connexin 43 gap junctions at the intercalated disc in individuals with LVH+SCD and in individuals with LVH + unnatural death (Control Group A) is similar. We observed, however, that there was an increase in the number of connexin 43–labeled gap junctions with an increase in the myocyte cross section. These gap junctions could potentially mediate conduction in the transverse orientation. By maintaining the expected number of gap junctions at the intercalated disc, the longitudinal conduction properties in myocardium of individuals with LVH would remain preserved, whereas the transverse conduction properties of the hypertrophied myocytes might be significantly altered.

### Limitations

This is a retrospective study performed in a small number of patients who sustained SCD or died of noncardiac causes and who underwent autopsy. Because we have relied on tissue from autopsy for histological characterization of myocardium, the individuals in this study comprise a highly selected group. Given these constraints, cases and controls were not matched for age, sex, or risk factors. The restricted sample size, especially in Control Group B, may have limited our ability to discern a statistically significant difference in normalized heart weight f Control Group A (LVH and death from noncardiac causes). Echocardiographic data was not available from all individuals included in this study, and therefore it is likely that not all individuals studied exhibited concentric LVH. Given these limitations, the observations presented in this study would need to be validated in a larger patient cohort and might need to be tested in other populations.

### Conclusions

We observed significant and specific alterations in the LV myocardial interstitium of individuals with isolated LVH and SCD, which may contribute to myocardial vulnerability for ventricular arrhythmias and warrant further mechanistic evaluation.
